# Special Issue: Present and Future Perspectives of Vascular Interventional Radiology

**DOI:** 10.3390/jpm13071131

**Published:** 2023-07-12

**Authors:** Julien Frandon, Jean-Paul Beregi

**Affiliations:** Department of Medical Imaging, IPI Plateform, Nîmes University Hospital, University of Montpellier, Medical Imaging Group Nîmes, IMAGINE, 30029 Nîmes, France; jean.paul.beregi@chu-nimes.fr

The field of vascular interventional radiology has witnessed remarkable advancements, transforming the landscape of patient care for both vascular and non-vascular pathologies. Through minimally invasive techniques, we can now offer therapeutic alternatives with reduced complications to patients who are not eligible for conventional treatments. With the emergence of personalized therapies tailored to specific pathologies, disease stages, and patient characteristics, we find ourselves at the forefront of a new era in medicine where treatments are uniquely tailored to each individual.

This Special Issue of the *Journal of Personalized Medicine* features a comprehensive collection of 16 articles, primarily focusing on embolization, a major theme in vascular interventional radiology. Embolization techniques have revolutionized the field, providing effective treatment options for various conditions. Within this issue, we delve into various aspects of embolization, including the embolization of the prostatic artery [1], splenic trauma [2], polycystic liver disease [3], soft tissue hematomas [4], pulmonary arteriovenous malformation [5], gastrointestinal bleeding [6], and even emerging themes such as musculoskeletal embolization [7]. These articles showcase the breadth and depth of research in the field, offering insights into the latest advancements and personalized approaches in embolization therapies.

One prominent focus of this issue revolves around prostatic artery embolization, an increasingly promising technique for treating benign prostatic hyperplasia. The articles within this issue present invaluable insights, offering tips and tricks to enhance the precision of the procedure using real-time navigation [8] devices and showcasing the benefits of radial access in facilitating outpatient management [9]. These techniques not only bolster the safety and efficiency of the procedure but also lead to reduced patient recovery times.

While embolization techniques have effectively addressed vascular pathologies, interventional radiology goes beyond vessel occlusion to include vessel recanalization. In cases of mesenteric ischemia, for instance, revascularization procedures have offered new avenues for treatment, restoring blood flow to ischemic organs and improving patient outcomes [10]. Furthermore, interventional radiologists have played a crucial role in enhancing vascularization by deploying stents in carotid arteries to improve blood supply to the brain [11,12]. Hence, interventional radiology not only occludes vessels but also serves as a powerful tool to reestablish perfusion, ultimately enhancing the overall management of various medical conditions.

In addition to its therapeutic applications, vascular interventional radiology is crucial in the diagnostic realm. By integrating the latest guidance technologies, such as cone beam CT, interventional radiologists can obtain detailed preoperative mapping for complex aortic surgeries [13]. New game-changing technologies such as 4DCT are helping to optimise patient exposure to X-rays for increasingly complex treatments [14]. In the same vein, this issue shows the contribution of MRI in diagnosing pelvic congestion syndrome, a condition often misdiagnosed or misunderstood, causing chronic pain and significant health challenges [15]. The synergy between advanced imaging techniques and interventional radiology empowers clinicians to make informed decisions, resulting in safer and more effective patient treatments.

Beyond vascular pathologies, several articles shed light on the application of vascular interventional radiology in treating non-vascular pathologies, including tendon pathology [7] and hepatocellular cancer [16]. These contributions underscore the importance of comprehending the underlying pathophysiology of these conditions and developing personalized therapeutic approaches.

Collectively, the articles published in this Special Issue of the *Journal of Personalized Medicine* underscore the pivotal role of vascular interventional radiology in contemporary clinical practice. These advances provide patients with innovative, minimally invasive treatment alternatives that reduce complications and enhance their overall quality of life. As personalized medicine continues to evolve, vascular interventional radiology is poised to play an increasingly significant role in delivering tailored treatments that cater to the specific requirements of individual patients.

In conclusion, we extend our gratitude to the authors for their valuable contributions to this Special Issue. Their research and insights hold immense potential to shape the future of vascular interventional radiology, driving the paradigm of personalized medicine. We encourage readers to delve into the articles and embrace the transformative power of innovative interventions in their pursuit of enhanced patient care.
